# Supplementary material for: Lexical semantic content, not syntactic structure, is the main contributor to ANN-brain similarity of fMRI responses in the language network
Source: bioRxiv. 2023 May 6:2023.05.05.539646. Preprint. [Version 1] doi: 10.1101/2023.05.05.539646 (PMC10187317; doi:10.1101/2023.05.05.539646)
Supplement: Supplement 1 [file NIHPP2023.05.05.539646v1-supplement-1.pdf]

bioRxiv preprint doi: https://doi.org/10.1101/2023.05.05.539646; this version posted May 6, 2023. The copyright holder for this preprint (which was not certified by peer review) is the author/funder, who has granted bioRxiv a license to display the preprint in perpetuity. It is made available under aCC-BY 4.0 International license.

Supplementary Information (SI)

Supplementary Figures

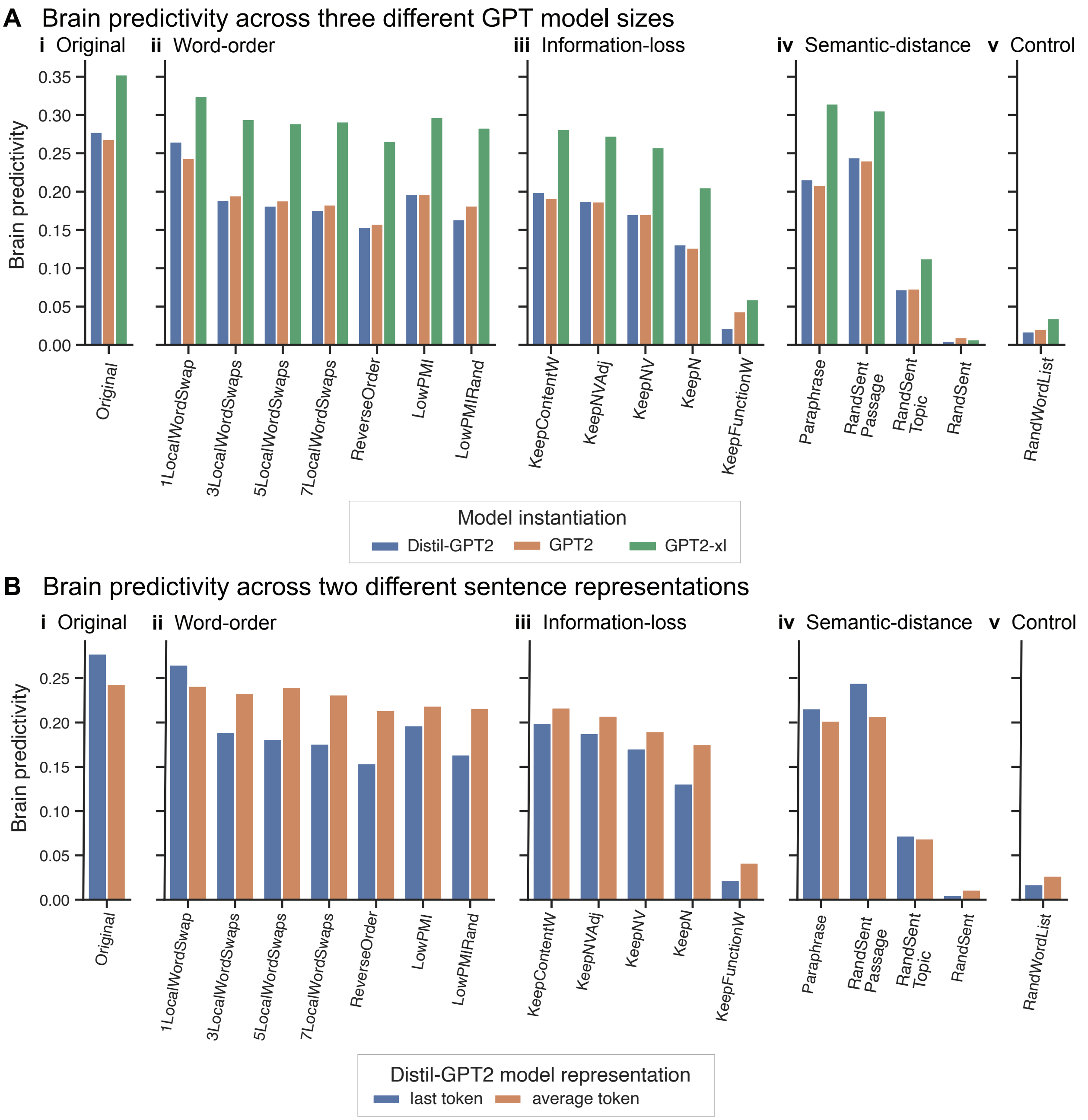

Figure SI 1. Robustness of brain predictivity scores across ANN model sizes and sentence representations. Across the three model instantiations and the two sequence summary versions, we observe a robust result pattern, with consistent numerical differences between models across conditions.

A. Predictivity performance of ANN-to-brain models trained on ANN representations of intact sentences and tested on ANN representations of perturbed sentences for three GPT-2 model instantiations (*TrainIntact-*

bioRxiv preprint doi: https://doi.org/10.1101/2023.05.05.539646; this version posted May 6, 2023. The copyright holder for this preprint (which was not certified by peer review) is the author/funder, who has granted bioRxiv a license to display the preprint in perpetuity. It is made available under aCC-BY 4.0 International license.

TestPerturbed_Contextualized): Distil-GPT2, GPT2, and GPT2-xl. **B.** Predictivity performance using two different approaches for representing the sentence for Distil-GPT2 (analyses performed using Distil-GPT2 due to computational cost and robust patterns across ANN model sizes, see panel A). The primary approach was the last token representation where the sequence representation is obtained at the last sentence token (see Methods; Retrieving ANN model representations). We investigated an alternative approach, the average token representation, where we computed the arithmetic mean of all the token representations in the sentence, excluding the token representations of the preceding sentences (if any): in particular, if a sentence was the second sentence within a passage, we did not take into account the token representations from the first sentence in that passage, only the tokens that comprise the current sentence.

bioRxiv preprint doi: https://doi.org/10.1101/2023.05.05.539646; this version posted May 6, 2023. The copyright holder for this preprint (which was not certified by peer review) is the author/funder, who has granted bioRxiv a license to display the preprint in perpetuity. It is made available under aCC-BY 4.0 International license.

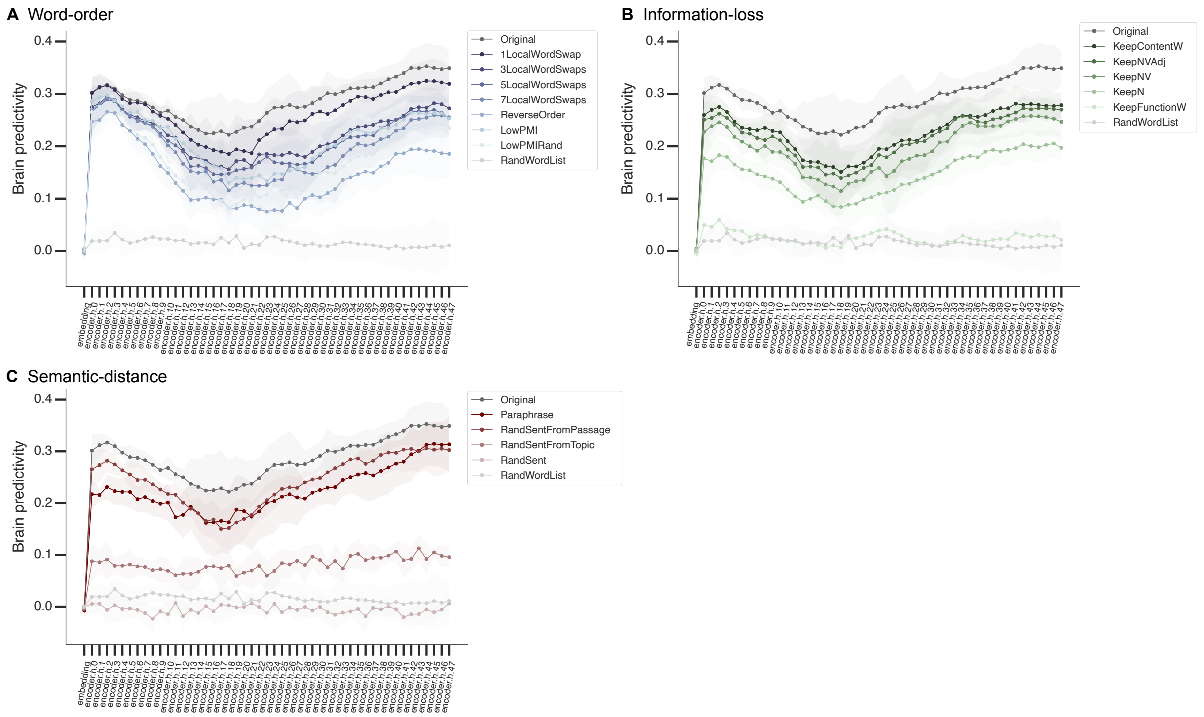

Figure SI 2. Brain predictivity scores across GPT2-xl layers for each perturbation manipulation condition. Each panel (A-C) shows the scores across layers for all perturbation manipulation conditions within each category. The mapping model was trained on ANN representations of intact sentences and evaluated on ANN representations of perturbed sentences (*TrainIntact-TestPerturbed_Contextualized*). The shaded regions illustrate the median absolute deviation (m.a.d.) error within participants.

bioRxiv preprint doi: https://doi.org/10.1101/2023.05.05.539646; this version posted May 6, 2023. The copyright holder for this preprint (which was not certified by peer review) is the author/funder, who has granted bioRxiv a license to display the preprint in perpetuity. It is made available under aCC-BY 4.0 International license.

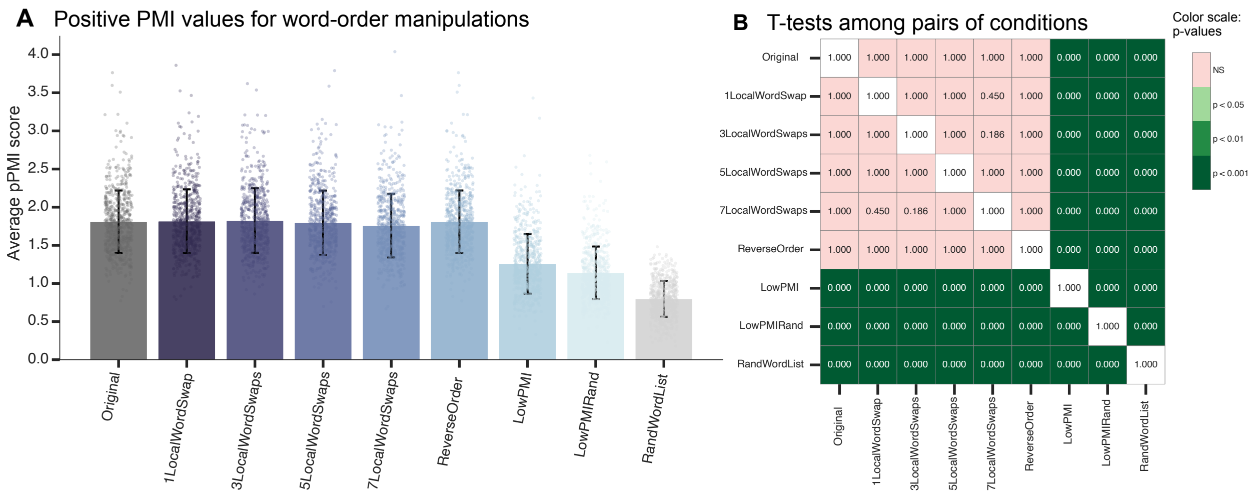

Figure SI 3. PMI verification of word-order manipulation conditions.

A. Average positive pointwise mutual information (PMI) for each word-order manipulation condition. Each data point represents a sentence, and the error bars show the standard deviation from the mean. The *{1,3,5,7}LocalWordSwaps* and *ReverseOrder* conditions were designed to preserve local dependency structure (see Methods; Perturbation manipulation conditions; Word-order manipulations). As expected, the *Original, {1,3,5,7}LocalWordSwaps* and *ReverseOrder* conditions were not significantly different from each other (panel B).

The two low-PMI conditions (*LowPMI* and *LowPMIRandom*) were designed to destroy local dependency structure. As expected, the deterministically created low-PMI condition (*LowPMI*), the nondeterministically-created low-PMI condition (*LowPMIRandom*) and the random wordlist condition (*RandWordList*) were each significantly different from all other conditions.

B. Significance was established via independent two-sided t-tests, with p-values corrected for multiple comparisons (within each perturbation manipulation condition) using the Bonferroni procedure, here shown as a grid of pairwise p-values for all comparisons.

bioRxiv preprint doi: https://doi.org/10.1101/2023.05.05.539646; this version posted May 6, 2023. The copyright holder for this preprint (which was not certified by peer review) is the author/funder, who has granted bioRxiv a license to display the preprint in perpetuity. It is made available under aCC-BY 4.0 International license.

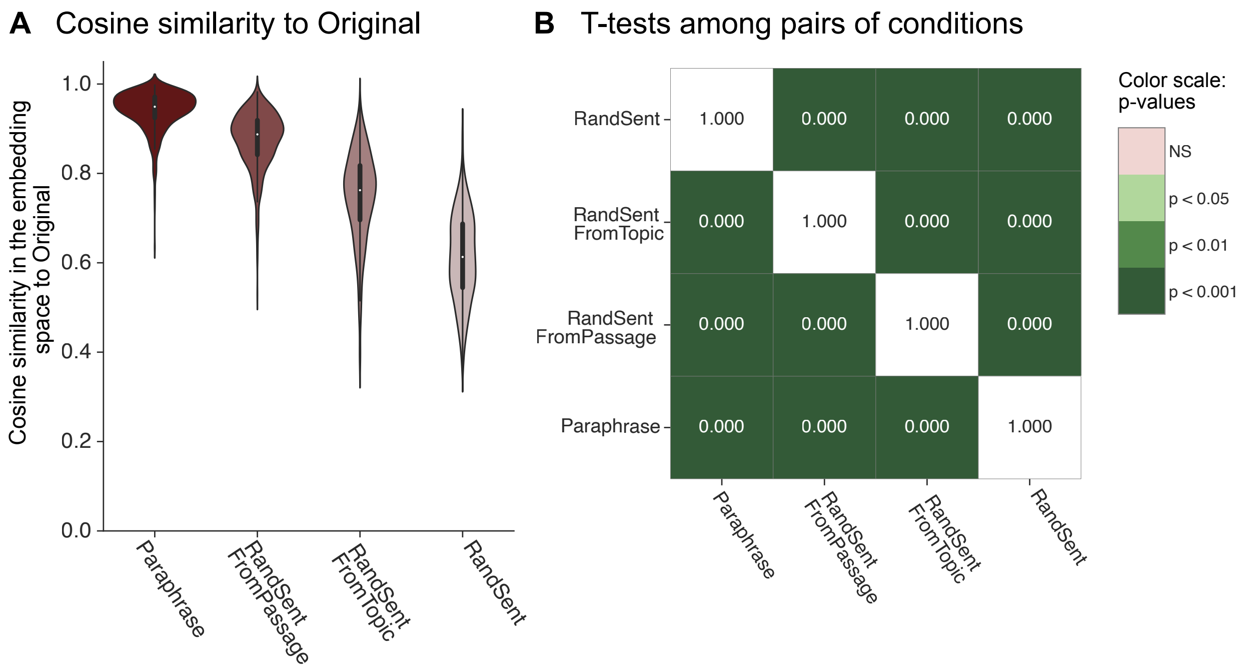

Figure SI 4. Validation of gradual semantic distance to Original within the semantic-distance perturbation conditions (quantified using contextualized embeddings from GPT2-xl).

A. We quantified the pairwise Cosine similarity of all 627 GPT2-xl sentence representation vectors for the *semantic-distance* manipulation datasets (conditions: *Paraphrase, RandSentFromPassage, RandSentFromTopic, RandSent*) with the representation of the intact version of the sentence (condition: *Original*). As expected, semantic similarity with the original sentence gradually decreased across conditions. **B.** The semantic-distance manipulations were significantly different from each other (independent two-sided t-tests with Bonferroni correction, ps<.001).

bioRxiv preprint doi: https://doi.org/10.1101/2023.05.05.539646; this version posted May 6, 2023. The copyright holder for this preprint (which was not certified by peer review) is the author/funder, who has granted bioRxiv a license to display the preprint in perpetuity. It is made available under aCC-BY 4.0 International license.

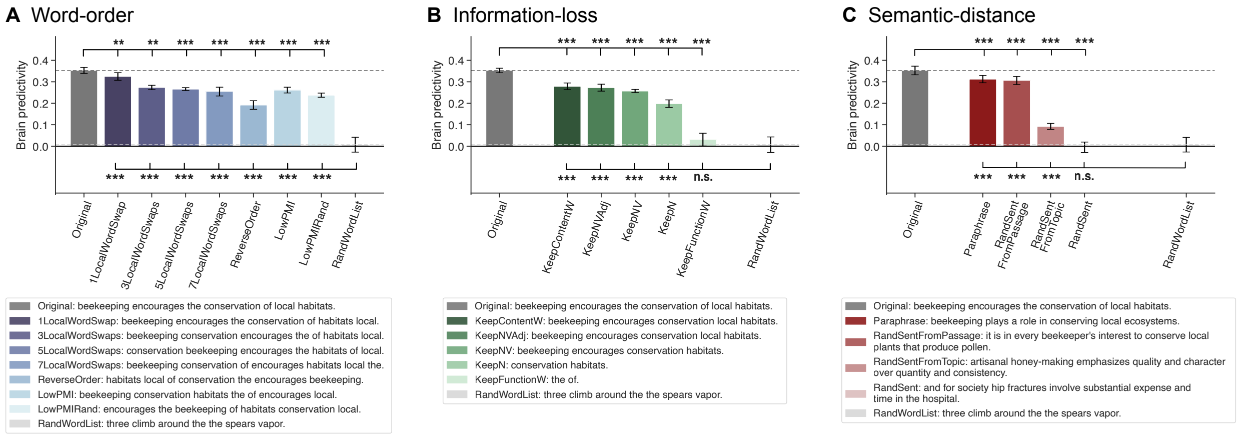

Figure SI 5. Brain predictivity performance of ANN-to-brain mapping models on held-out sentences using a fixed layer across all perturbation manipulation conditions (as opposed to the best-performing layer for each condition as shown in Figure 2 in the main text).

As in Figure 2, the mapping model was trained on ANN representations of intact sentences and evaluated on ANN representations of perturbed sentences (*TrainIntact-TestPerturbed_Contextualized*). Different from Figure 2, for this analysis, we selected the layer that performed best on the *Original* benchmark (encoder layer 44) instead of selecting the best-performing layer per condition. Bars show the brain predictivity using this fixed layer across the three perturbation manipulation conditions. Note that the *RandWordList* control condition reaches chance-level performance (zero).

bioRxiv preprint doi: https://doi.org/10.1101/2023.05.05.539646; this version posted May 6, 2023. The copyright holder for this preprint (which was not certified by peer review) is the author/funder, who has granted bioRxiv a license to display the preprint in perpetuity. It is made available under aCC-BY 4.0 International license.

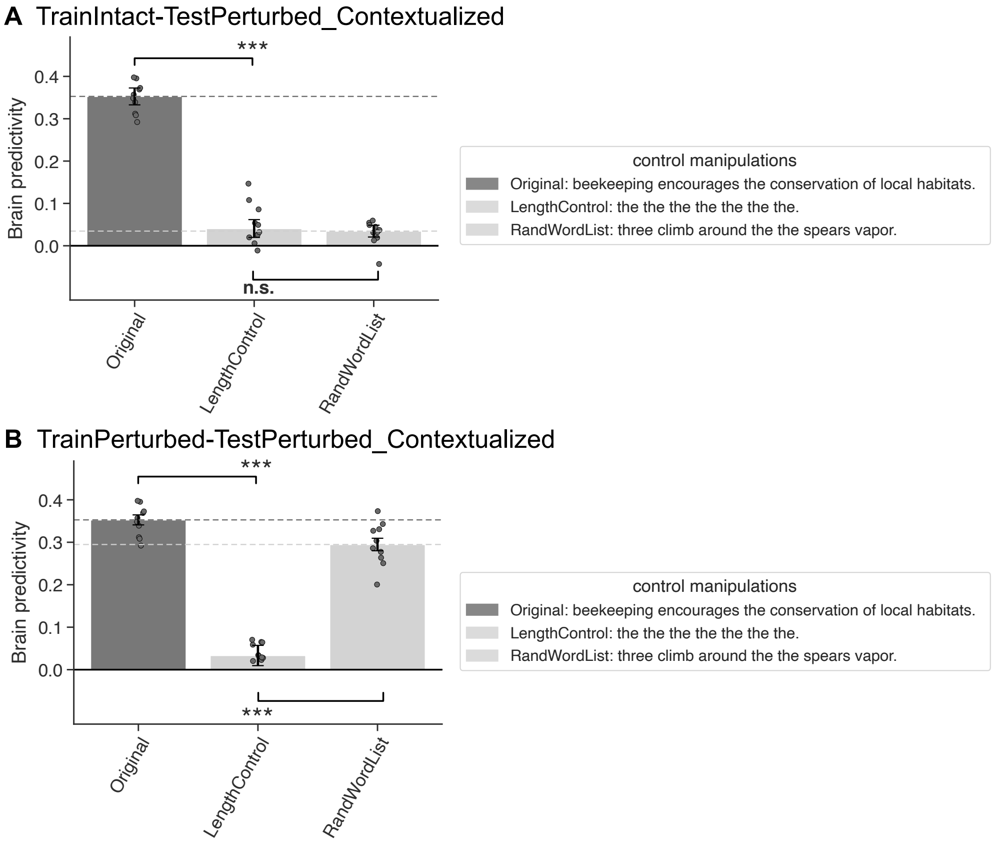

Figure SI 6. A length-controlled benchmark (*LengthControl*) performs on par with random word lists (*RandWordList*), and performs significantly better than chance.

A. Performance of the ANN-to-brain mapping model on held-out sentences, trained on ANN representations of intact sentences and evaluated on ANN representations of perturbed sentences (*TrainIntact-TestPerturbed_Contextualized*) on an additional control condition *LengthControl* (each word in the original sentence replaced by the word "the", allowing to test for effects due to the number of words in the sentence), relative to *Original* and *RandWordList*. A one-sample t-test shows that the *LengthControl* condition leads to non-zero predictivity performance ($t=3.34$, $p<.01$).
B. Performance of ANN-to-brain mapping model on held-out sentences, trained on ANN representations of perturbed sentences and evaluated on ANN representations of perturbed sentences (*TrainPerturbed-TestPerturbed_Contextualized*), including the control condition *LengthControl*. A one-sample t-test shows that the *LengthControl* condition leads to non-zero predictivity performance ($t=6.84$, $p<.001$).

bioRxiv preprint doi: https://doi.org/10.1101/2023.05.05.539646; this version posted May 6, 2023. The copyright holder for this preprint (which was not certified by peer review) is the author/funder, who has granted bioRxiv a license to display the preprint in perpetuity. It is made available under aCC-BY 4.0 International license.

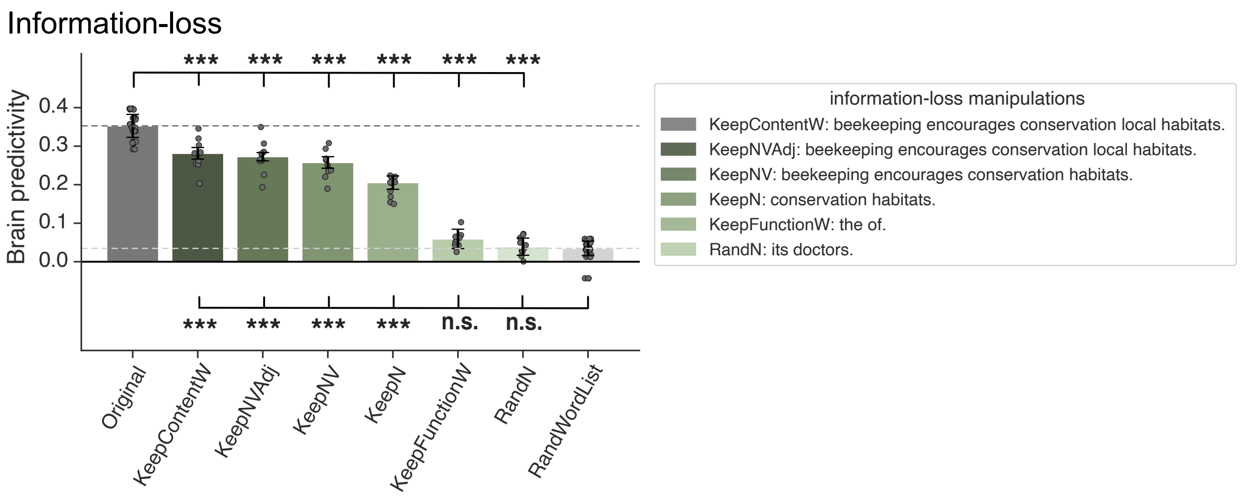

Figure SI 7. A benchmark with random nouns (*RandN*) performs on par with random word lists (*RandWordList*). The additional control benchmark, *RandN*, contained exclusively nouns (randomly sampled from the nouns in the dataset) and was matched for length with the KeepN condition. Brain predictivity performance of ANN-to-brain mapping model on held-out sentences (*TrainPerturbed-TestPerturbed_Contextualized*) of the *RandN* benchmark along with the remaining information-loss manipulations. The *RandN* benchmark performed on par with the random word list benchmark, *RandWordList*.

bioRxiv preprint doi: https://doi.org/10.1101/2023.05.05.539646; this version posted May 6, 2023. The copyright holder for this preprint (which was not certified by peer review) is the author/funder, who has granted bioRxiv a license to display the preprint in perpetuity. It is made available under aCC-BY 4.0 International license.

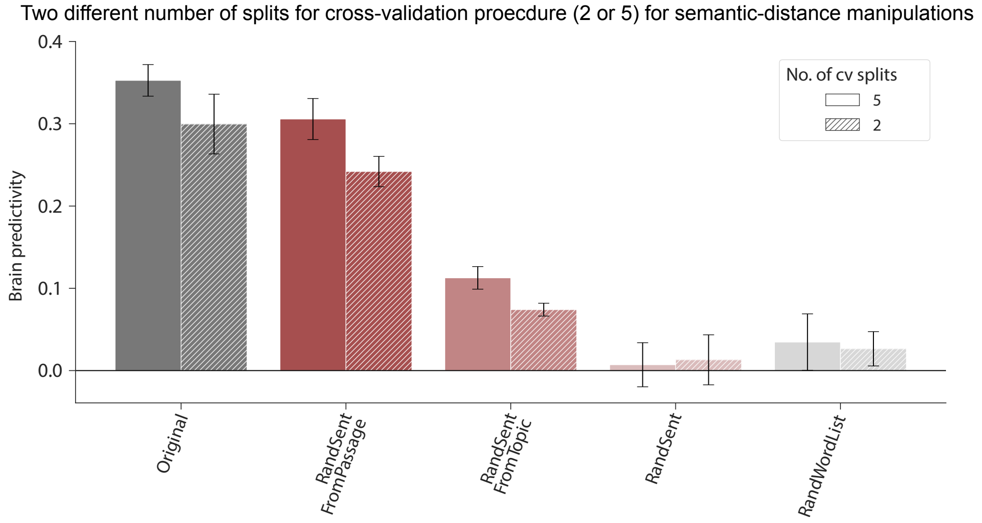

Figure SI 8. Key patterns of results are not affected by choice of number of splits in cross-validation procedure. Brain predictivity performance of ANN-to-brain mapping model on held-out sentences (*TrainIntact-TestPerturbed_Contextualized*) of the semantic-distance perturbation manipulation category benchmarks which were designed to shuffle sentences within the dataset (see Methods; Perturbation manipulation conditions; Semantic-distance manipulations). Due to the infeasibility of assigning sentences to 5 cross-validation folds and shuffling sentences according to the hierarchical structure of the Pereira et al. (2018) dataset, we additionally ran the *RandSent, RandSentFromPassage and RandSentFromTopic TrainIntact-TestPerturbed* benchmark versions (along with the *Original* and *RandWordList* benchmarks for comparison) using only 2 cross-validation splits instead of the default number of 5-folds. Using this procedure, all but 17.17% of sentence representations could be shuffled relative to its associated fMRI data for *RandSentFromPassage* and all sentences could be successfully shuffled with the associated fMRI data for *RandSentFromTopic*, leading to a less biased benchmark compared to the default 5-fold cross-validation scheme. The key patterns of results were not affected. For consistency with the remaining results, the 5-fold cross-validation results are reported in the main text.

bioRxiv preprint doi: https://doi.org/10.1101/2023.05.05.539646; this version posted May 6, 2023. The copyright holder for this preprint (which was not certified by peer review) is the author/funder, who has granted bioRxiv a license to display the preprint in perpetuity. It is made available under aCC-BY 4.0 International license.

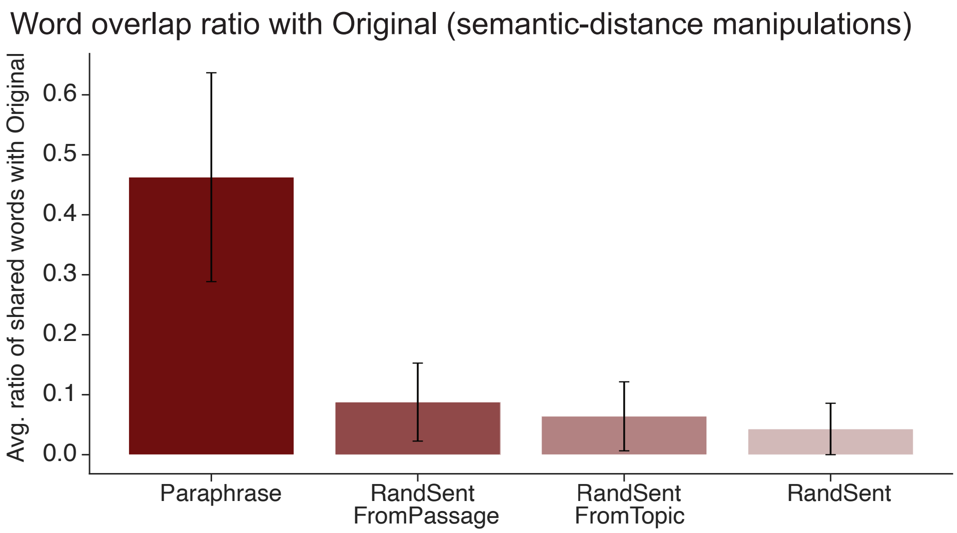

Figure SI 9. Quantification of word overlap ratio with *Original* across semantic-distance manipulations. For each of the semantic-distance manipulations, we quantified the ratio of word overlap for each sentence with the corresponding sentence from the *Original* condition and averaged the overlap ratios to obtain a summary statistic. Word overlap was quantified as (unique number of overlapping words) / (unique number of words in ({*semantic-distance manipulation*} + *Original*)). Error bars show the standard deviation from the mean.

bioRxiv preprint doi: https://doi.org/10.1101/2023.05.05.539646; this version posted May 6, 2023. The copyright holder for this preprint (which was not certified by peer review) is the author/funder, who has granted bioRxiv a license to display the preprint in perpetuity. It is made available under aCC-BY 4.0 International license.

Brain predictivity vs. degree of similarity in the embedding space across computational designs

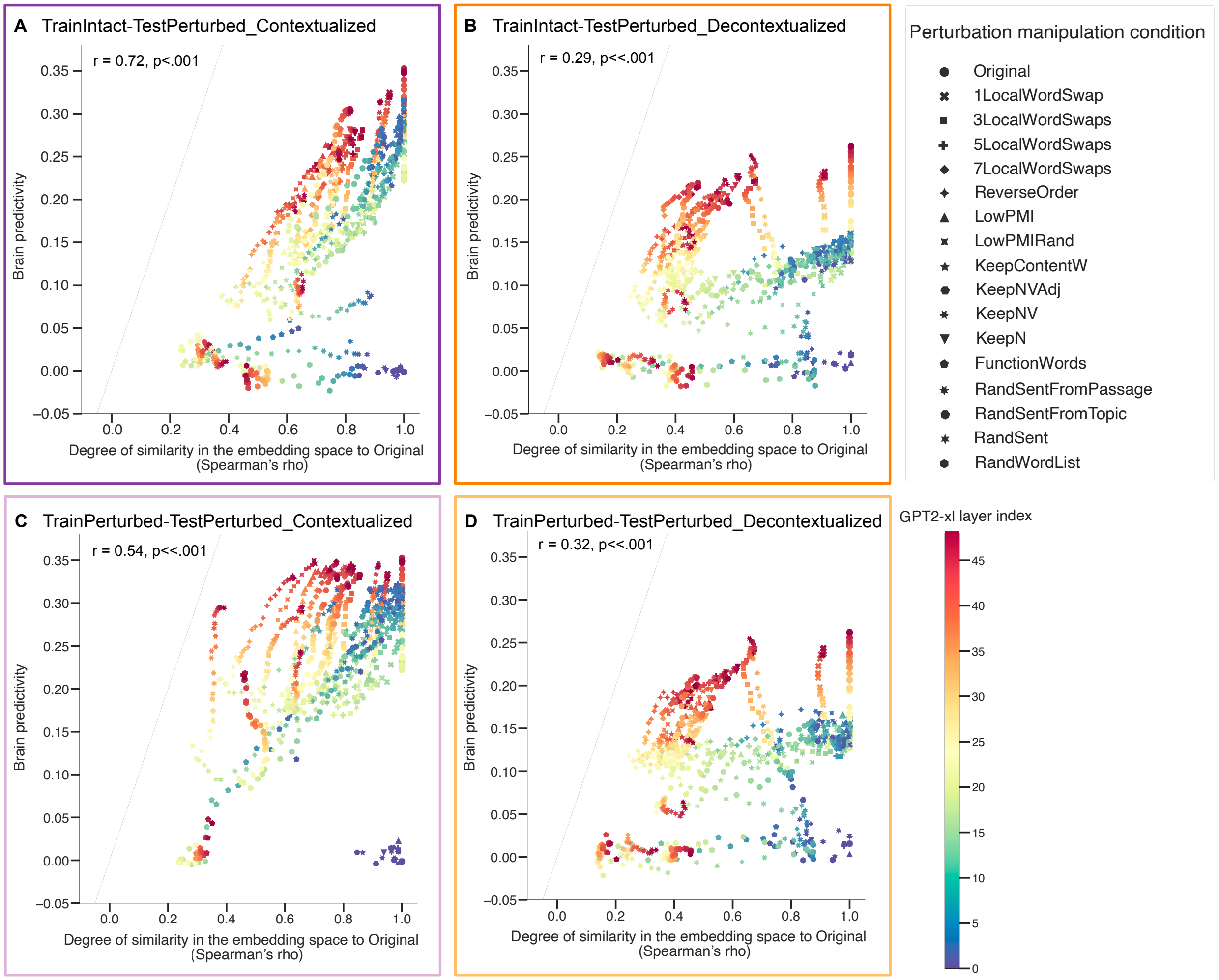

Figure SI 10. Representational similarity to the original sentences is correlated with brain predictivity (across all computational experimental designs).

Each individual data point shows the correlation between brain predictivity (y-axis) and degree of similarity to the intact sentence set (x-axis, quantified using the Spearman's rank correlation coefficient, ρ) for a layer of the GPT2-xl ANN model and a certain perturbation manipulation condition for all computational experimental designs (panels A-D). The ANN layer index is denoted by colors. The perturbation manipulation condition is denoted by data point marker symbols.

bioRxiv preprint doi: https://doi.org/10.1101/2023.05.05.539646; this version posted May 6, 2023. The copyright holder for this preprint (which was not certified by peer review) is the author/funder, who has granted bioRxiv a license to display the preprint in perpetuity. It is made available under aCC-BY 4.0 International license.

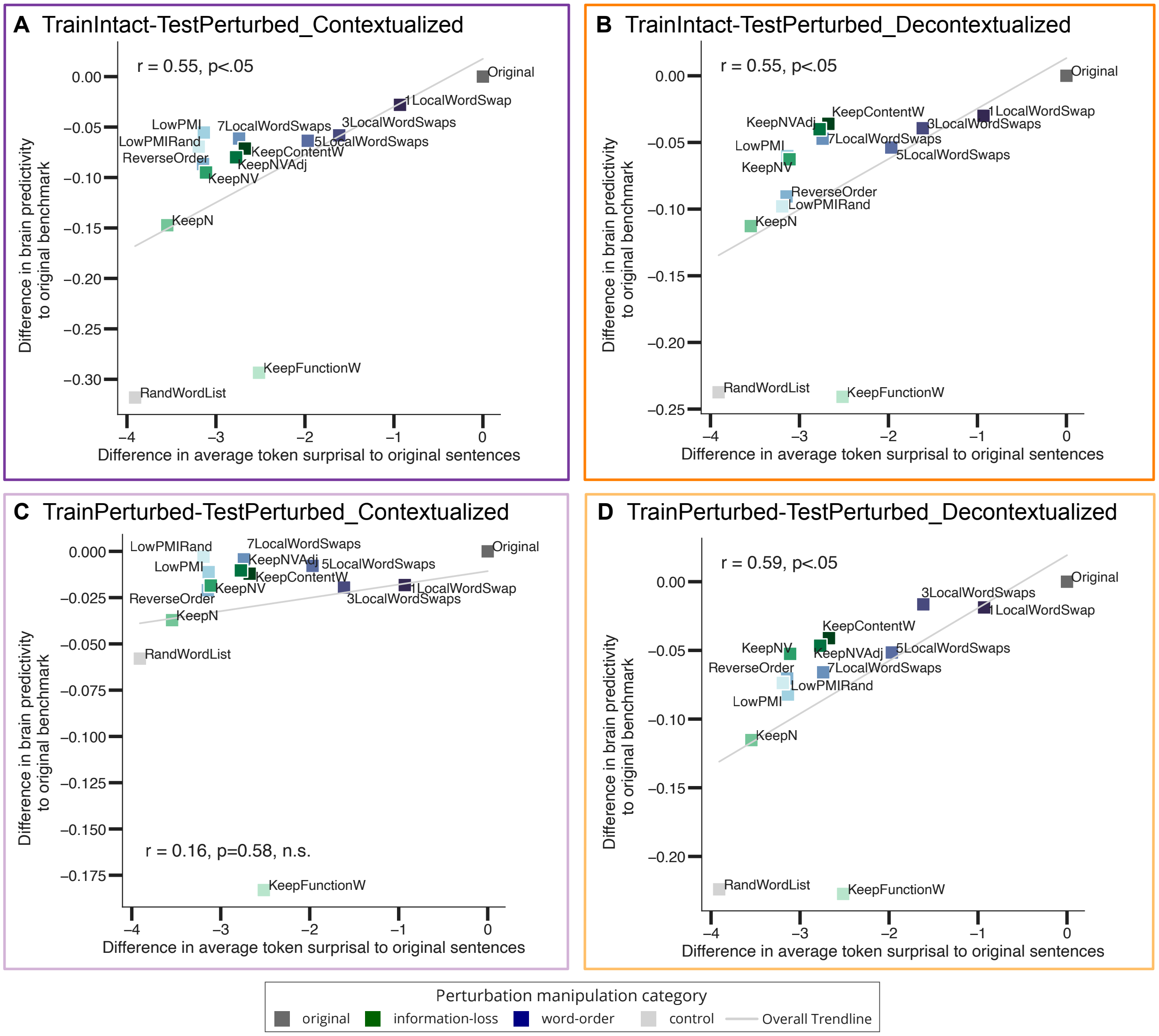

Figure SI 11. Perturbation manipulations that render sentences on average more surprising lead to lower brain predictivity (across all computational experimental designs).

The plots show the correlation between i) the difference in average sentence token surprisal between each perturbed sentence set and the original sentence set and ii) the difference in brain predictivity scores between each perturbed benchmark and the *Original* benchmark across all computational experimental designs (panels A-D).

bioRxiv preprint doi: https://doi.org/10.1101/2023.05.05.539646; this version posted May 6, 2023. The copyright holder for this preprint (which was not certified by peer review) is the author/funder, who has granted bioRxiv a license to display the preprint in perpetuity. It is made available under aCC-BY 4.0 International license.

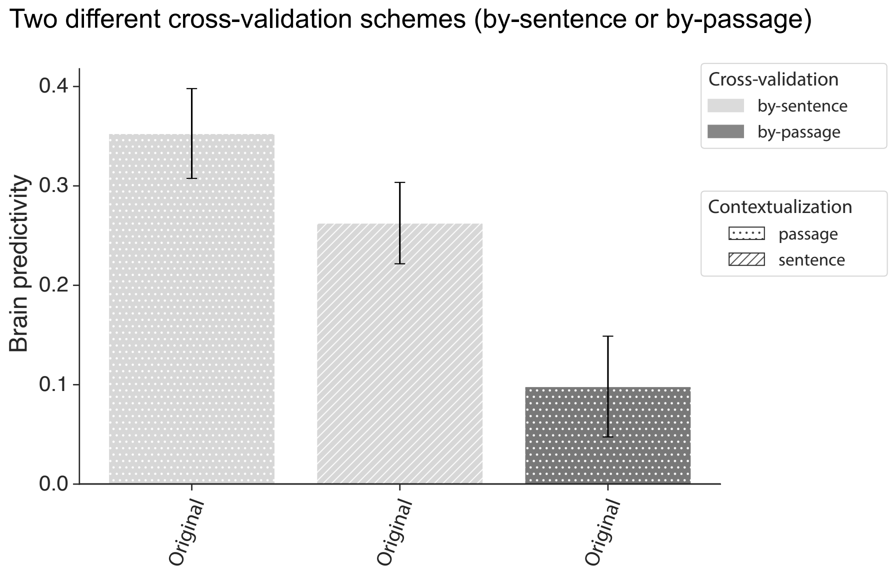

Figure SI 12. Brain predictivity performance of ANN-to-brain mapping using two different cross-validation schemes.
Stimuli were separated into train and test sets in two ways: i) by sentence, disregarding the passage structure in the Pereira et al. 2018 dataset (light gray bars) vs. ii) by passage, such that all the sentences from the same passage end up in the same set (train or test) rather than being split across those sets (dark gray bar). For comparison to Results; Section 3.3, we show the contextualized and decontextualized ANN representation in the by-sentence split scheme as investigated throughout the manuscript. The ANN-to-brain mapping model was canonically trained (i.e., trained on the *Original,* intact sentences and tested on intact sentences (*TrainPerturbed-TestPerturbed_Contextualized*), consistent with prior work). Error bars show median absolute deviation (m.a.d.) error within participants.

bioRxiv preprint doi: https://doi.org/10.1101/2023.05.05.539646; this version posted May 6, 2023. The copyright holder for this preprint (which was not certified by peer review) is the author/funder, who has granted bioRxiv a license to display the preprint in perpetuity. It is made available under aCC-BY 4.0 International license.

Supplementary Tables

Example of passage from Experiment 2 (384 sentences, 96 passages). Topic: Musical Instrument.

**1**	An accordion is a portable musical instrument with two keyboards.
**2**	One keyboard is used for individual notes, the other for chords.
**3**	Accordions produce sound with bellows that blow air through reeds.
**4**	An accordionist plays both keyboards while opening and closing the bellows.

Example of passage from Experiment 3 (243 sentences, 72 passages). Topic: Beekeeping.

**1**	Beekeeping encourages the conservation of local habitats.
**2**	It is in every beekeeper's interest to conserve local plants that produce pollen.
**3**	As a passive form of agriculture, it does not require that native vegetation be cleared to make way for crops.
**4**	Beekeepers also discourage the use of pesticides on crops, because they could kill the honeybees.

Table SI 1. Examples of passages from Experiment 2 and Experiment 3 from Pereira et al. (2018), respectively.

bioRxiv preprint doi: https://doi.org/10.1101/2023.05.05.539646; this version posted May 6, 2023. The copyright holder for this preprint (which was not certified by peer review) is the author/funder, who has granted bioRxiv a license to display the preprint in perpetuity. It is made available under aCC-BY 4.0 International license.

	**Comparison with *Original***			**Comparison with *RandWordList***		
	**T-test statistic**	**Adjusted p-value**	**Cohen's d**	**T-test statistic**	**Adjusted p-value**	**Cohen's d**
1LocalWordSwap	5.517	0.0026	0.572	-18.327	0.0000	-8.288
3LocalWordSwaps	5.814	0.0018	1.527	-17.437	0.0000	-9.487
5LocalWordSwaps	6.507	0.0008	1.630	-17.104	0.0000	-9.322
7LocalWordSwaps	6.781	0.0006	1.641	-16.790	0.0000	-9.230
ReverseOrder	9.285	0.0000	2.383	-15.265	0.0000	-8.060
LowPMI	6.354	0.0009	1.560	-20.505	0.0000	-9.994
LowPMIRand	8.467	0.0001	2.043	-17.035	0.0000	-8.542
KeepContentW	9.209	0.0000	1.855	-19.649	0.0000	-7.141
KeepNVAdj	12.825	0.0000	1.977	-16.826	0.0000	-6.617
KeepNV	17.944	0.0000	2.697	-16.811	0.0000	-6.942
KeepN	28.583	0.0000	4.842	-13.441	0.0000	-5.743
KeepFunctionW	17.400	0.0000	9.866	-2.571	0.1506	-1.122
Paraphrase	10.782	0.0000	1.238	-17.966	0.0000	-8.251
RandSentFromPassage	6.843	0.0003	0.975	-17.600	0.0000	-8.621
RandSentFromTopic	28.347	0.0000	7.502	-6.438	0.0005	-2.654
RandSent	24.262	0.0000	11.849	2.088	0.2657	1.002

Table SI 2. Statistics for Figure 2 in the main text (Results; Section 3.1). Pairwise, two-sided, dependent t-tests for all comparisons performed between the condition *Original* and all conditions of interest, as well as between condition *RandWordList* and all conditions of interest across perturbation manipulation classes. P-values were corrected for multiple comparisons (within each perturbation manipulation condition) using the Bonferroni procedure. Effect sizes, as quantified by Cohen's d are reported.

bioRxiv preprint doi: https://doi.org/10.1101/2023.05.05.539646; this version posted May 6, 2023. The copyright holder for this preprint (which was not certified by peer review) is the author/funder, who has granted bioRxiv a license to display the preprint in perpetuity. It is made available under aCC-BY 4.0 International license.

Condition 1	Condition 2	T-test statistic	Adjusted p-value	Significance	Cohen's d	Manipulation
1LocalWordSwap	3LocalWordSwaps	2.9	0.369	n.s.	0.77	word-order
1LocalWordSwap	5LocalWordSwaps	3.469	0.148	n.s.	0.867	word-order
1LocalWordSwap	7LocalWordSwaps	3.437	0.156	n.s.	0.882	word-order
1LocalWordSwap	ReverseOrder	6.886	0.002	**	1.588	word-order
1LocalWordSwap	LowPMI	3.568	0.127	n.s.	0.771	word-order
1LocalWordSwap	LowPMIRand	5.775	0.006	**	1.267	word-order
3LocalWordSwaps	5LocalWordSwaps	3.3	0.194	n.s.	0.127	word-order
3LocalWordSwaps	7LocalWordSwaps	3.076	0.278	n.s.	0.15	word-order
3LocalWordSwaps	ReverseOrder	6.27	0.003	**	1.055	word-order
3LocalWordSwaps	LowPMI	-0.161	1.0	n.s.	-0.032	word-order
3LocalWordSwaps	LowPMIRand	3.849	0.082	n.s.	0.648	word-order
5LocalWordSwaps	7LocalWordSwaps	0.403	1.0	n.s.	0.024	word-order
5LocalWordSwaps	ReverseOrder	6.046	0.004	**	0.929	word-order
5LocalWordSwaps	LowPMI	-0.898	1.0	n.s.	-0.165	word-order
5LocalWordSwaps	LowPMIRand	3.185	0.233	n.s.	0.523	word-order
7LocalWordSwaps	ReverseOrder	5.07	0.014	*	0.9	word-order
7LocalWordSwaps	LowPMI	-0.927	1.0	n.s.	-0.189	word-order
7LocalWordSwaps	LowPMIRand	2.599	0.604	n.s.	0.496	word-order
ReverseOrder	LowPMI	-7.609	0.001	**	-1.137	word-order
ReverseOrder	LowPMIRand	-2.491	0.721	n.s.	-0.4	word-order
LowPMI	LowPMIRand	3.82	0.086	n.s.	0.711	word-order
**Condition 1**	**Condition 2**	**T-test statistic**	**Adjusted p-value**	**Significance**	**Cohen's d**	**Manipulation**
KeepContentW	KeepNVAdj	1.224	1.0	n.s.	0.185	information-loss
KeepContentW	KeepNV	5.581	0.003	**	0.684	information-loss
KeepContentW	KeepN	9.939	0.0	***	2.516	information-loss
KeepContentW	KeepFunctionW	13.773	0.0	***	7.019	information-loss
KeepNVAdj	KeepNV	3.167	0.114	n.s.	0.461	information-loss
KeepNVAdj	KeepN	10.063	0.0	***	2.186	information-loss
KeepNVAdj	KeepFunctionW	11.576	0.0	***	6.413	information-loss
KeepNV	KeepN	9.472	0.0	***	1.923	information-loss
KeepNV	KeepFunctionW	12.889	0.0	***	6.861	information-loss
KeepN	KeepFunctionW	9.804	0.0	***	5.597	information-loss
**Condition 1**	**Condition 2**	**T-test statistic**	**Adjusted p-value**	**Significance**	**Cohen's d**	**Manipulation**
Paraphrase	RandSentFromPassage	-1.476	1.0	n.s.	-0.271	semantic-distance
Paraphrase	RandSentFromTopic	22.931	0.0	***	6.089	semantic-distance
Paraphrase	RandSent	20.345	0.0	***	10.255	semantic-distance
RandSentFromPassage	RandSentFromTopic	20.488	0.0	***	6.45	semantic-distance
RandSentFromPassage	RandSent	24.504	0.0	***	10.713	semantic-distance
RandSentFromTopic	RandSent	8.25	0.0	***	4.231	semantic-distance

bioRxiv preprint doi: https://doi.org/10.1101/2023.05.05.539646; this version posted May 6, 2023. The copyright holder for this preprint (which was not certified by peer review) is the author/funder, who has granted bioRxiv a license to display the preprint in perpetuity. It is made available under aCC-BY 4.0 International license.

Table SI 3. Additional Statistics for Figure 2 in the main text (Results; Section 3.1). Pairwise, two-sided, dependent t-tests for all comparisons performed between the conditions within the same perturbation manipulation classes. P-values were corrected for multiple comparisons (within each perturbation manipulation condition) using the Bonferroni procedure. Effect sizes, as quantified by Cohen's d, are reported.

bioRxiv preprint doi: https://doi.org/10.1101/2023.05.05.539646; this version posted May 6, 2023. The copyright holder for this preprint (which was not certified by peer review) is the author/funder, who has granted bioRxiv a license to display the preprint in perpetuity. It is made available under aCC-BY 4.0 International license.

Manipulation type	Condition	Average length (in words)
Original	*Original*	12.33
Information-loss	*KeepContentW*	8.20
	KeepNVAdj	7.79
	KeepNV	6.70
	KeepN	4.35
	RandN	4.35
	KeepFunctionW	4.13

Table SI 4. Overview of average sentence length across information-loss manipulation conditions (*Original* condition included for reference). Note that the conditions in all other perturbation manipulation classes have the same number of words as the Original condition.

bioRxiv preprint doi: https://doi.org/10.1101/2023.05.05.539646; this version posted May 6, 2023. The copyright holder for this preprint (which was not certified by peer review) is the author/funder, who has granted bioRxiv a license to display the preprint in perpetuity. It is made available under aCC-BY 4.0 International license.

Experimental Design 1	Experimental Design 2	T-test statistic	Adjusted p-value	Significance	Cohen's d	Manipulation
TrainIntact-TestPerturbed_Contextualized	TrainIntact-TestPerturbed_Decontextualized	12.761	0.0	***	2.724	original
TrainIntact-TestPerturbed_Contextualized	TrainPerturbed-TestPerturbed_Contextualized	NaN	NaN	n.s.	0.0	original
TrainIntact-TestPerturbed_Contextualized	TrainPerturbed-TestPerturbed_Decontextualized	12.779	0.0	***	2.725	original
TrainIntact-TestPerturbed_Decontextualized	TrainPerturbed-TestPerturbed_Contextualized	-12.761	0.0	***	-2.724	original
TrainIntact-TestPerturbed_Decontextualized	TrainPerturbed-TestPerturbed_Decontextualized	1.71	0.728	n.s.	0.001	original
TrainPerturbed-TestPerturbed_Contextualized	TrainPerturbed-TestPerturbed_Decontextualized	12.779	0.0	***	2.725	original
TrainIntact-TestPerturbed_Contextualized	TrainIntact-TestPerturbed_Decontextualized	24.965	0.0	***	2.6	word-order
TrainIntact-TestPerturbed_Contextualized	TrainPerturbed-TestPerturbed_Contextualized	-11.522	0.0	***	-1.203	word-order
TrainIntact-TestPerturbed_Contextualized	TrainPerturbed-TestPerturbed_Decontextualized	21.512	0.0	***	2.441	word-order
TrainIntact-TestPerturbed_Decontextualized	TrainPerturbed-TestPerturbed_Contextualized	-26.873	0.0	***	-3.547	word-order
TrainIntact-TestPerturbed_Decontextualized	TrainPerturbed-TestPerturbed_Decontextualized	-3.217	0.012	*	-0.239	word-order
TrainPerturbed-TestPerturbed_Contextualized	TrainPerturbed-TestPerturbed_Decontextualized	25.058	0.0	***	3.428	word-order
TrainIntact-TestPerturbed_Contextualized	TrainIntact-TestPerturbed_Decontextualized	12.249	0.0	***	0.643	information-loss
TrainIntact-TestPerturbed_Contextualized	TrainPerturbed-TestPerturbed_Contextualized	-13.436	0.0	***	-1.025	information-loss
TrainIntact-TestPerturbed_Contextualized	TrainPerturbed-TestPerturbed_Decontextualized	8.364	0.0	***	0.565	information-loss
TrainIntact-TestPerturbed_Decontextualized	TrainPerturbed-TestPerturbed_Contextualized	-24.0	0.0	***	-1.76	information-loss
TrainIntact-TestPerturbed_Decontextualized	TrainPerturbed-TestPerturbed_Decontextualized	-1.298	1.0	n.s.	-0.074	information-loss
TrainPerturbed-TestPerturbed_Contextualized	TrainPerturbed-TestPerturbed_Decontextualized	24.699	0.0	***	1.662	information-loss
TrainIntact-TestPerturbed_Contextualized	TrainIntact-TestPerturbed_Decontextualized	5.916	0.0	***	0.358	semantic-distance
TrainIntact-TestPerturbed_Contextualized	TrainPerturbed-TestPerturbed_Contextualized	-7.058	0.0	***	-1.109	semantic-distance
TrainIntact-TestPerturbed_Contextualized	TrainPerturbed-TestPerturbed_Decontextualized	5.972	0.0	***	0.422	semantic-distance
TrainIntact-TestPerturbed_Decontextualized	TrainPerturbed-TestPerturbed_Contextualized	-13.32	0.0	***	-1.942	semantic-distance
TrainIntact-TestPerturbed_Decontextualized	TrainPerturbed-TestPerturbed_Decontextualized	1.632	0.664	n.s.	0.073	semantic-distance
TrainPerturbed-TestPerturbed_Contextualized	TrainPerturbed-TestPerturbed_Decontextualized	15.065	0.0	***	2.059	semantic-distance
TrainIntact-TestPerturbed_Contextualized	TrainIntact-TestPerturbed_Decontextualized	0.468	1.0	n.s.	0.135	control
TrainIntact-TestPerturbed_Contextualized	TrainPerturbed-TestPerturbed_Contextualized	-12.789	0.0	***	-6.414	control

bioRxiv preprint doi: https://doi.org/10.1101/2023.05.05.539646; this version posted May 6, 2023. The copyright holder for this preprint (which was not certified by peer review) is the author/funder, who has granted bioRxiv a license to display the preprint in perpetuity. It is made available under aCC-BY 4.0 International license.

TrainIntact-TestPerturbed_Contextualized	TrainPerturbed-TestPerturbed_Decontextualized	-0.05	1.0	n.s.	-0.014	control
TrainIntact-TestPerturbed_Decontextualized	TrainPerturbed-TestPerturbed_Contextualized	-12.437	0.0	***	-6.767	control
TrainIntact-TestPerturbed_Decontextualized	TrainPerturbed-TestPerturbed_Decontextualized	-0.519	1.0	n.s.	-0.151	control
TrainPerturbed-TestPerturbed_Contextualized	TrainPerturbed-TestPerturbed_Decontextualized	13.808	0.0	***	6.421	control

Table SI 5. Statistics for Figure 4 in the main text (Results; Section 3.3). Pairwise, two-sided, dependent t-tests for all comparisons performed between the computational experimental design conditions across perturbation manipulation classes. P-values were corrected for multiple comparisons (within each perturbation manipulation condition) using the Bonferroni procedure.

bioRxiv preprint doi: https://doi.org/10.1101/2023.05.05.539646; this version posted May 6, 2023. The copyright holder for this preprint (which was not certified by peer review) is the author/funder, who has granted bioRxiv a license to display the preprint in perpetuity. It is made available under aCC-BY 4.0 International license.

Supplementary Methods

Estimation of ceiling. Due to intrinsic noise in biological measurements, we estimated a ceiling value (i.e., a normalizing constant for the correlation between actual and ANN-predicted brain responses) which quantifies how well the best possible "average human" model could perform on predicting brain responses in single voxels for held-out "target" participants. In our ceiling estimation, we included the n=5 participants that completed both experiments in the Pereira et al. (2018) dataset to obtain full overlap in the materials across participants. Following Schrimpf et al. (2021), the ceiling value was estimated using a three-step procedure:

<u>Step 1: Collect data for extrapolation</u>

We first subsampled the data with n=5 recorded participants into all possible combinations of p participants for all $p \in [2, n=5]$. For example, for p=2 there are ${}_5P_2=20$ possible subsample combinations from the participant pool. To keep computational cost manageable, for each subsample size p, we used only 10 of these random subsample combinations to calculate correlation scores (that will be used in the extrapolation in Step 2). For example, for p=2, we randomly picked 10 subsamples from the 10 possible participant subsamples of size 2 from the 5 participants in the participant pool. For each of these subsamples for a given p, we then designated one participant as the target (such that each participant is chosen as the target once) whose brain responses we attempt to predict from the remaining p-1 participants (e.g., predict 1 participant from 1 (other) participant (p=2), 1 from 2 participants (p=3), …, 1 from n-1 participants (p=n)) to obtain a correlation score between the predicted and the actual activation of the voxel for each voxel in the "target" participant for the given subsample. Hence, instead of predicting a "target" participant's voxels using an ANN embedding (as done in the main analysis), we used voxels from the "predictor" participants as an embedding (if only one participant was used as the "predictor" participant, we used that participant's voxels; if two or more participants were used as "predictor" participants, their voxels were concatenated).

<u>Step 2: Get ceiling value per voxel by extrapolating prediction accuracy to infinitely many participants</u>

We computed a ceiling value for each voxel individually using an extrapolation approach. To extrapolate the approach described in Step 1 beyond the number of participants in the participant pool (n=5), we fitted the equation for each voxel where p is each subsample's number of participants (i.e., 2, …, 5), v is each subsample's correlation score and are the fitted parameters for asymptote and slope respectively. For each voxel, we used 100 bootstraps to fit the ceiling to different subsets of predicted values across subsamples and used the median of the asymptote values from the 100 bootstraps as that voxel's ceiling value. Specifically, for each of the 100 bootstraps, we resample the correlation values (with replacement) for each p and fit the equation above.

<u>Step 3: Aggregate the ceiling values across voxels to obtain the ceiling value</u>

After estimating a ceiling value for all voxels in all of the 5 participants as described in Steps 1 and 2, we used these scores to compute the dataset's final ceiling value that was used as a

bioRxiv preprint doi: https://doi.org/10.1101/2023.05.05.539646; this version posted May 6, 2023. The copyright holder for this preprint (which was not certified by peer review) is the author/funder, who has granted bioRxiv a license to display the preprint in perpetuity. It is made available under aCC-BY 4.0 International license.

normalizing constant for the brain predictivity scores (correlation between actual and ANN-predicted brain responses). To do so, we computed the median of the per-voxel ceilings across all voxels and all participants. Via this procedure, we obtained a final ceiling value of 0.32 for the Pereira et al. (2018) dataset. The model scores we report are the model's overall raw correlation scores (aggregated as described in Methods; Comparison of ANN model representations to brain measurements) divided by this ceiling value.
